# Worsening Paraparesis: A Diagnostic Dilemma for Neurosarcoidosis

**DOI:** 10.7759/cureus.25958

**Published:** 2022-06-15

**Authors:** Alexandra Stroia, Shista Priyadarshini, Marcelle Meseeha

**Affiliations:** 1 Internal Medicine, Guthrie Robert Packer Hospital, Sayre, USA; 2 Internal Medicine, Lake Erie College of Osteopathic Medicine, Erie, USA

**Keywords:** atypical presentation of sarcoidosis, neurosarcoidosis, sarcoidosis, thalamic lesion, paraplegia

## Abstract

Sarcoidosis is a chronic granulomatous disorder mostly known to affect the respiratory system. However, about 5-10% of cases develop neurological complications, either de novo or in patients with known sarcoidosis. The most common complications as cited by current literature include cranial nerve palsies, meningitis, and myelopathy. A unilateral thalamic lesion is an extremely rare presentation of disease. As the neurological manifestations of sarcoidosis are uncommon and variable, it poses a diagnostic challenge. We present a challenging case with worsening paraparesis and a step-by-step approach to how it was diagnosed as neurosarcoidosis. We aimed to create awareness about this uncommon manifestation to avoid misdiagnosis and promote early recognition of neurosarcoidosis.

## Introduction

Sarcoidosis is a chronic granulomatous disease of an unknown etiology and is known to affect multiple organ systems. In approximately 70% of cases, it is known to affect the respiratory system [1]. The other 30% of cases have extrapulmonary manifestations [2]. Approximately 5-10% cases of sarcoidosis develop neurologic manifestations [3]. Neurosarcoidosis is an uncommon manifestation that presents either with neurologic complaints in known sarcoidosis or de novo [4]. Diagnosis is often challenging as any component of the nervous system can be affected and symptoms are diverse. This article aimed to highlight an atypical presentation of previously undiagnosed neurosarcoidosis and its stepwise approach to diagnosis. We also want to create awareness among the medical fraternity about this uncommon and puzzling diagnosis.

## Case presentation

A 45-year-old white woman presented with complaints of worsening lower extremity weakness for the last two months. Her past medical history was significant for asthma, chronic sinusitis, diabetes mellitus type 2, migraine, polycystic ovarian syndrome, and depression. During detailed history, the patient reported slowing of her gait dating back to five years which recently worsened to proximal lower extremity weakness over the last two months. Over the years, she had multiple complaints ranging from malaise, fatigue, multiple joint aches, and nonspecific pain which was labeled as fibromyalgia. She continued to have on and off flares of musculoskeletal pain.

Meanwhile, she continued to have a myriad of symptoms including new-onset intermittent slurring of speech, numbness, tingling, intermittent tinnitus, vertigo, bilateral worsening deafness, and numbness of right side of the face. The patient underwent neurological examination which was unremarkable except for an incidental Arnold-Chiari I malformation on MRI head with and without contrast. The patient had progressive sensorineural deafness that continued to worsen along with progressive tinnitus resulting in complete deafness in left ear on the day of presentation. Audiological evaluation showed sensorineural deafness in bilateral ears, left>right. She was started on galcanezumab for better symptomatic control for vestibular migraine. Episodic vertigo and dizziness continued and she was presumed to have basilar migraine. Two months prior to presentation, she reported a new onset headache and bilateral predominantly proximal lower extremity weakness. Neurological and rheumatological evaluations were unremarkable.

This new onset frontal headache, usually in evenings associated with nausea was evaluated during this admission. On presentation, she was hypertensive 160/98, afebrile, and saturating well on room air. Physical examination showed no abnormality in respiratory, cardiac, or abdominal system. The patient was slow to respond but alert and oriented to time, place, and person. The patient was left-sided dominant. Neurological examination revealed strength in extremities as right upper 4/5, left upper extremity 5/5, right lower 3/5, and left lower 4/5. There were no sensory deficits and reflexes were grade 2 throughout. She was also noted to have left-sided deafness but no deficits in vision or in cranial nerves II-VII and IX-XII. The patient denied any recent tick exposure.

**Figure 1:**
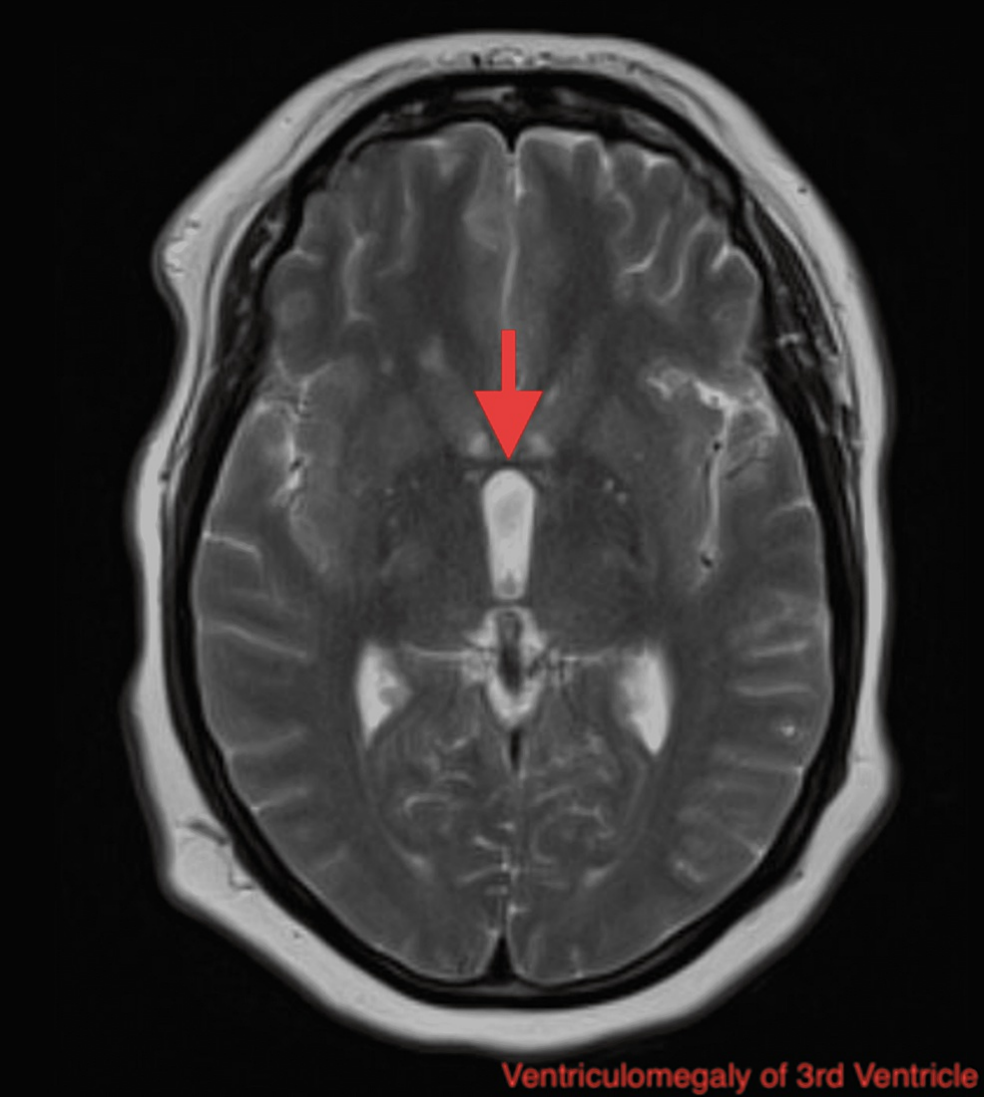
MRI of the head showing new ventriculomegaly of the third ventricle (arrow indicating hydrocephalus).

**Figure 2:**
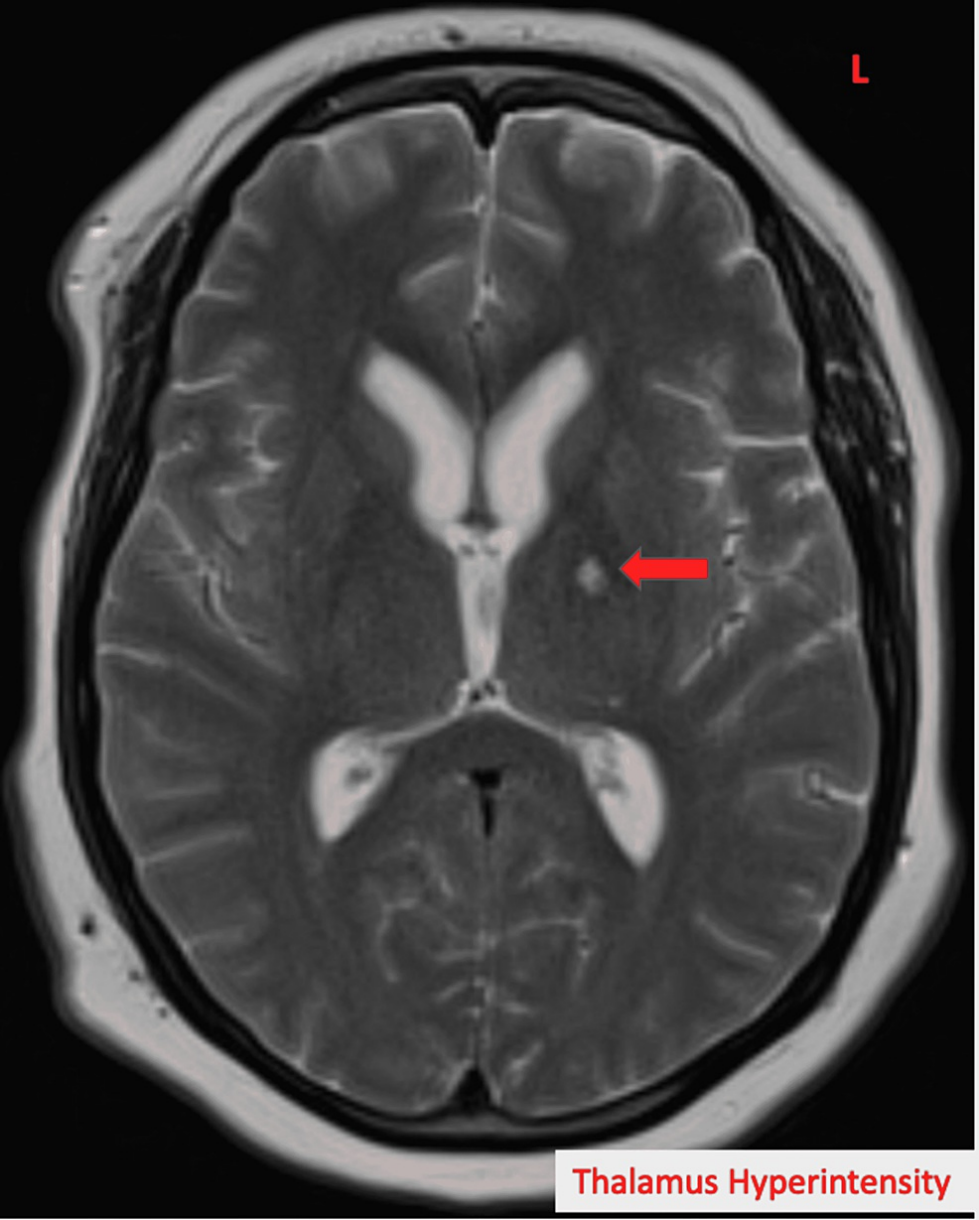
MRI brain showing hyperintensity of the left thalamus in T2 (arrow indicates the lesion).

**Figure 3:**
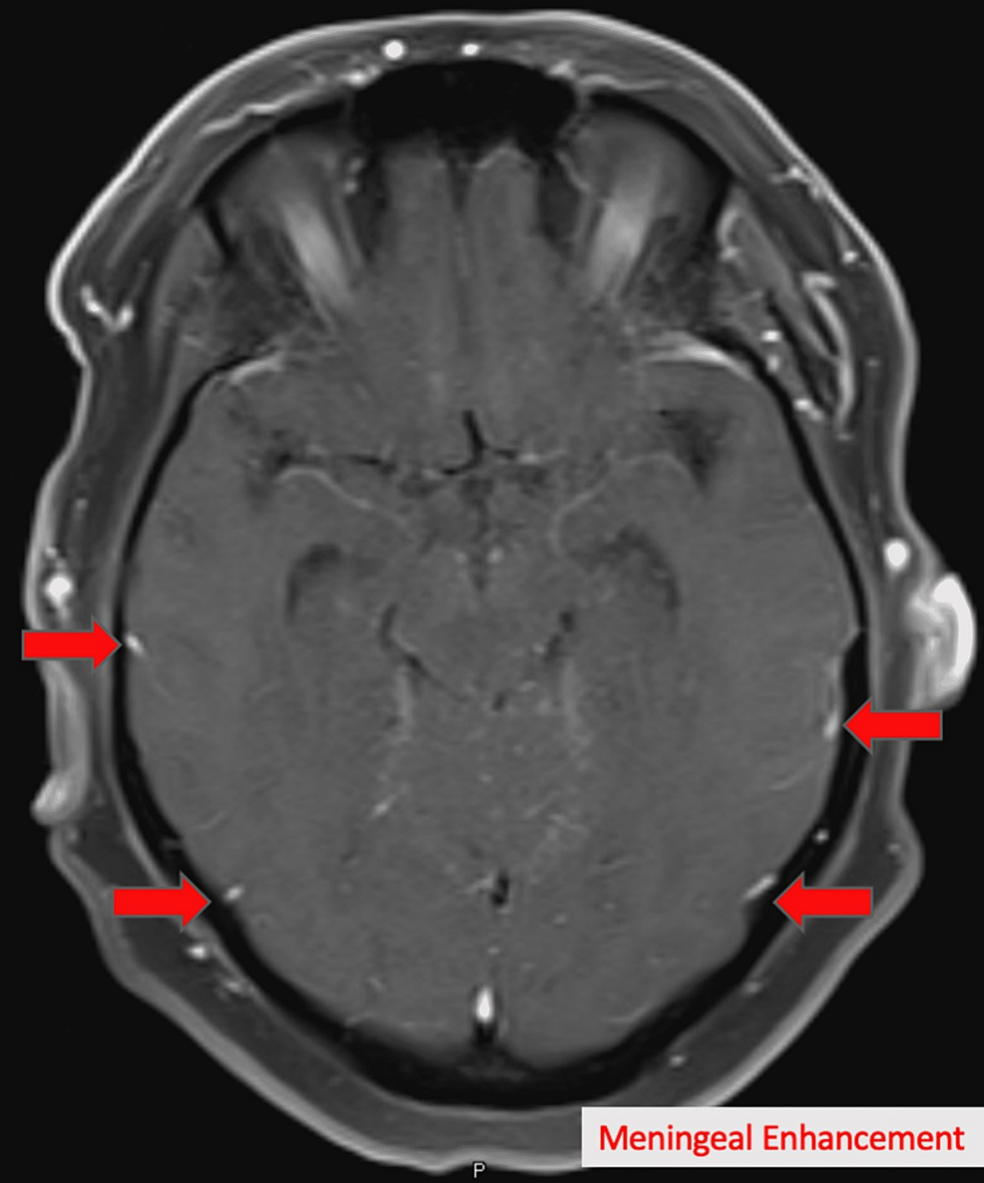
MRI of head showing new onset meningeal enhancement (arrows indicating enhancements).

Laboratory investigations showed elevations in erythrocyte sedimentation rate (ESR) 63 (nl < 30 mm/h) and C-reactive protein (CRP) 2.20 (nl < 0.5 mg/dL). Rheumatologic workup with anti-histone, antinuclear (ANA), antiribonucleoprotein(anti-RNP), anti-RNA polymerase III markers was inconclusive for any diagnosis. Some skin nodules were noted however they did not seem indicative of erythema nodosum. Lyme disease panel was negative as well. MRI brain showed new ventriculomegaly (Figure 1), increased T2 signal in the left thalamus (Figure 2), and meningeal enhancement when compared to previous studies (Figure 3). 

 

Lumbar puncture showed an opening pressure of 35 cm of H_2_O (Table 1). Cerebrospinal fluid studies demonstrated nucleated cells 87 with 92% lymphocytes, low glucose 22, and elevated protein level 241 (Table 1). Oligoclonal bands were absent (Table 1). Infectious workup was done for cerebrospinal fluid (CSF) pleocytosis.

**Table 1:** Cerebrospinal fluid findings.

	Patient	Normal
Opening pressure	35	7-18 mmH_2_O
Protein	241	23-38 mg/dL
Glucose	22	45-85 mg/dL
White blood cell count	87	< 5 cells/mm^3^
Cell differential	92% lymphocytes 8% monocytes 0% eosinophils	0-5 cells
Oligoclonal bands	Negative	Negative
Gram stain/culture	Negative	Negative
Bartonella IgM	Negative	Negative
Anaplasma IgM	Negative	Negative
Herpes simplex virus IgM	Negative	Negative

Laboratory investigations showed ESR 49, CRP 1.20, WBC 8.73, neutrophils 74.4%, hemoglobin 15.1, and total protein 7.1 (Table 2). Blood and CSF culture showed no growth. Bartonella, anaplasma and herpes simplex virus immunoglobulin M were negative. Serological studies for systemic diseases including rheumatoid factor, lupus, Sjogren, and antiphospholipid antibodies showed no abnormalities (Table 2). Heavy metal screen was negative as well. With no conclusive diagnosis, there was concern for paraneoplastic neuropathy. Paraneoplastic panel anti-Hu, anti-CRMP5, anti-GAD65, anti-NR1 were negative (Table 2).

**Table 2:** Laboratory findings of the patient. CRMP5: collapsin response mediator protein 5; GAD65: glutamic acid decarboxylase 65; NR: nuclear receptor 1

	Patient	Normal
White blood cells	8.73	4.0-10.5 x 10^9^/L
Neutrophils	74.4	30-75%
Hemoglobin	15.1	13.5-17.5 g/dL
Erythrocyte sedimentation rate	49	< 30 mm/h
C-reactive protein	1.20	< 0.5 mg/dL
Total protein	7.1	6.4-8.3 g/dL
Anti-histone antibody	Negative	Negative
Anti-nuclear antibody	Negative	Negative
Anti-RNA polymerase III	Negative	Negative
Rheumatoid factor	Negative	Negative
Anti-phospholipid antibody	Negative	Negative
Anti-Hu antibody	Negative	Negative
Anti-CRMP5 antibody	Negative	Negative
Anti-GAD65 antibody	Negative	Negative
Anti-NR1 antibody	Negative	Negative

**Figure 4:**
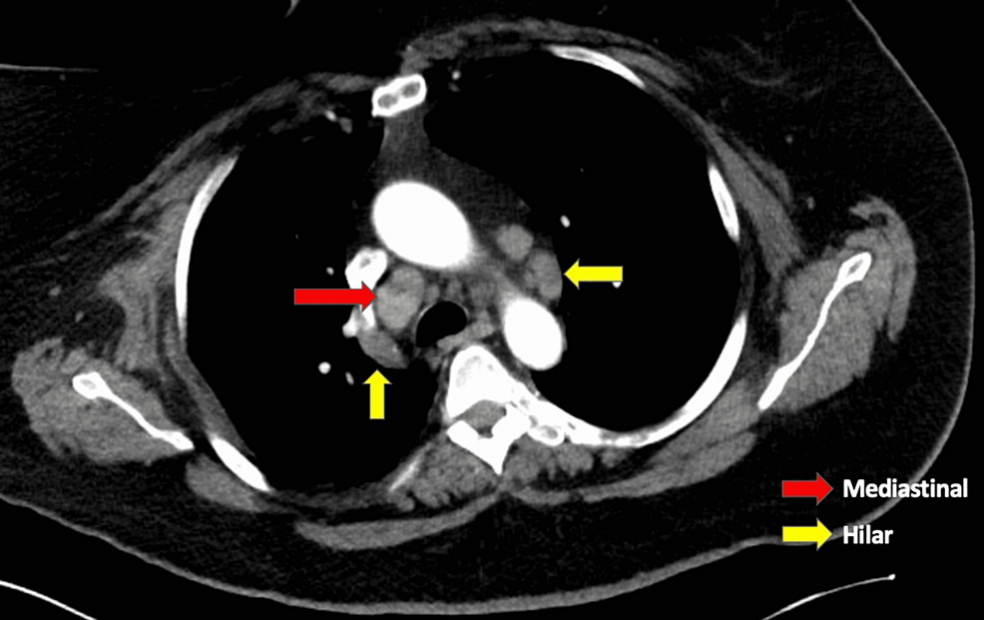
CT scan of the chest revealed mediastinal and hilar lymphadenopathy (red arrow indicating mediastinal nodes; yellow arrows indicating hilar nodes).

A computed tomography scan of the chest revealed mediastinal and hilar lymphadenopathy raising differentials for ​​lymphoproliferative disease, lymphoma, sarcoidosis, and infection (Figure 4). An endobronchial ultrasound was used to perform a transbronchial biopsy of the mediastinal lymphadenopathy. The biopsy revealed noncaseating granulomas. After correlating with the clinical picture and current literature, she was finally diagnosed with neurosarcoidosis.

## Discussion

Neurosarcoidosis is a lesser-known and uncommon presentation of sarcoidosis. Sarcoidosis is a granulomatous disorder affecting multiple organ systems with varying disease presentations and radiologic findings which make the diagnosis challenging [5,6]. It is known to commonly affect the lung along with other organs including lymph nodes, liver, eyes, and skin [7]. Neurological involvement is rare, occurring in only 5-10% of patients. In about 50% of total cases of neurosarcoidosis, the neurological complaints are usually the first to appear, making it difficult to diagnose. According to a meta-analysis of 1088 patients with neurosarcoidosis, around 50-70% cases will take the form of central and peripheral nervous system involvement [8]. The central lesions commonly present with headaches, disturbances in vision, auditory dysfunction, and various cranial nerve involvement with facial nerve being the most commonly affected [9]. Along with these, focal neurologic deficits have been reported in the setting of generalized seizures and encephalopathy, as well as spinal cord lesions leading to radiculopathies and myelopathies [10]. The skull base is the most common location to be affected and identified with non-caseating granulomas on biopsy [11].

Our patient had a unique presentation with unilateral thalamic lesion leading to worsening paraparesis. Rare cases of acute unilateral syndromes, mostly due to strokes involving the anterior or posterior‐lateral parts of the thalamus have been reported [12-14]. In these cases, afferent sensory, vestibular, and/or oculomotor pathway defects were described. And unlike our patient, these cases reported complete to near complete recovery of postural deficits within days to weeks.

The diagnostic criteria for central nervous system sarcoidosis and peripheral nervous system sarcoidosis were published by Stern et al. in a study by the Neurosarcoidosis Consortium Consensus Group [11]. This study defined possible, probable, and definite neurosarcoidosis. Each included a prerequisite of clinical and diagnostic evaluations (MRI, cerebrospinal fluid {CSF} studies, electromyogram, or nerve conduction study findings) typical of granulomatous inflammation after exclusion of other causes. Contrast-enhanced MRI is the preferred imaging modality for granulomatous disease as it allows for monitoring of disease progression [15]. Our patient had evidence of neurologic system involvement as demonstrated by slowing of gait, slurring of speech, paraparesis of lower extremities, and bilateral worsening deafness with MRI showing an increased T2 signal in the left thalamus. Systemic involvement was identified on transbronchial biopsy which revealed noncaseating granulomas. As other diseases were excluded, our patient therefore met criteria for probable neurosarcoidosis.

In cases where tissue biopsy of central nervous system lesions cannot be obtained the diagnosis is clinically deduced through contrast-enhanced MRI, lumbar puncture, laboratory testing, and detection of systemic sarcoidosis in other organs [6]. Basilar, diffuse, or nodular leptomeningeal enhancement on MRI has been reported in about 30-40% of cases [16]. CSF findings that occurred in 50% of neurosarcoidosis patients include elevated angiotensin converting enzyme, lymphocytic or neutrophilic pleocytosis, elevated total protein level, and reduced or normal glucose [17]. Oligoclonal bands and soluble CSF interleukin-2 receptors may also be increased. Our patient had increased nucleated cells with 92% lymphocytes, low glucose 22, and elevated protein level 241, without any oligoclonal bands on serology.

Corticosteroids are considered the mainstay of treatment and are hypothesized to provide benefit through anti-inflammatory and immuno-modulating effects [15]. However, the ability to achieve complete remission has been reported in fewer than one-third of patients with permanent neurological sequelae of neurosarcoidosis [18]. In cases where spinal cord involvement is present, second-line treatment involving immunosuppressive therapy or third-line treatment involving monoclonal antibodies has been reported to improve symptomatology [19]. Ultimately due to the vast systemic effects of disease a multidisciplinary approach involving neurologists, neurosurgeons, pulmonologists, rheumatologists, and ophthalmologists is usually required [20].

## Conclusions

Neurosarcoidosis is a rare entity accounting for almost 5-10% of all cases of sarcoidosis. In about half of the neurosarcoidosis cases, neurological complaints are the first presentation. Physicians should be aware of this rare condition and proper workup should be done. Neurosarcoidosis should be included in differential diagnosis and should be considered after proper exclusion of other associated conditions.
